# Modified Root Canal Sealers: A Comprehensive Review of Bioactive Enhancements, Antimicrobial Strategies, and Physicochemical Performance

**DOI:** 10.7759/cureus.114747

**Published:** 2026-08-18

**Authors:** Pauline Susan Palose, Helly D Thaker, Madonna Beskalis, Rozina Ali, Priya T Singh, Batoul Elsayed

**Affiliations:** 1 Department of Conservative Dentistry and Endodontics, Vinayaka Mission’s Sankarachariyar Dental College, Salem, IND; 2 Department of Dental Surgery, Narsinhbhai Patel Dental College and Hospital, Visnagar, IND; 3 Department of Endodontics, Faculty of Dentistry, Cairo University, Giza, EGY; 4 Department of Dental Surgery, Baqai Dental College, Karachi, PAK; 5 Department of Dental Surgery, Sri Rajiv Gandhi College of Dental Sciences and Hospital, Bengaluru, IND; 6 Department of Dental Surgery, Badr University in Cairo, Badr, EGY

**Keywords:** antimicrobial activity, bioactive materials, bioceramic sealers, nanoparticles, physicochemical properties, root canal sealers

## Abstract

Root canal sealers are essential for achieving a three-dimensional seal of the root canal system, thereby preventing reinfection and facilitating periapical healing. Despite decades of material development, no available sealer satisfies every property expected of an ideal material.

This review examines the rationale for modifying conventional sealers, focusing on recurring shortcomings including solubility, cytotoxicity, dimensional instability, and limited bioactivity. Recent work has concentrated on calcium silicate-based and bioceramic chemistries and on the incorporation of nanoparticles, bioactive glass, quaternary ammonium compounds, chitosan, and metal oxides to improve antimicrobial activity, physicochemical stability, and biological function. Experimental modifications generally enhanced antimicrobial activity, ion release, remineralization potential, or biological performance. However, their effects on flow, solubility, dimensional stability, cytocompatibility, and retreatability were formulation- and concentration-dependent, and clinical evidence remains limited. Persistent challenges, particularly retreatability and the shortage of long-term clinical validation, continue to justify further research toward optimized multifunctional endodontic sealers.

## Introduction and background

Root canal sealers occupy a central role in obturation, working with the core filling material to establish a fluid-tight closure, reduce the risk of reinfection, and create an environment favorable to periapical repair [1]. No material presently in clinical use meets all the criteria of an ideal sealer, which has driven continuous refinement of established formulations [2]. Modification is motivated by the need to improve physical and chemical stability, antimicrobial performance, and bioactivity, and to simplify clinical handling while limiting cytotoxicity and solubility [3]. Conventional zinc oxide-eugenol (ZOE) and epoxy resin sealers provide acceptable sealing but carry limitations, including shrinkage, incomplete adaptation to dentin, cytotoxic constituents, and absence of bioactivity, any of which can undermine long-term outcomes.

An ideal root canal sealer should exhibit dimensional stability, low solubility in tissue fluids, strong adhesion to both dentin and the core filling material, adequate radiopacity, antimicrobial activity, excellent biocompatibility, and the ability to be removed efficiently during retreatment procedures [2]. These requirements can conflict because a compositional change that improves one property may compromise another. The survival of organisms such as *Enterococcus faecalis* within dentinal tubules and isthmuses further increases the functional demands on sealers, which may constitute the final barrier between residual microorganisms and periradicular tissues [4]. This review therefore describes the rationale for sealer modification, the principal modification strategies, and their physicochemical, biological, and clinical implications.

In this context, obturation refers to the filling and sealing of the prepared root canal system with a core material and sealer to minimize pathways for microbial re-entry. Bioactivity refers to the ability of a material to interact favorably with the surrounding biological environment, including ion release, apatite formation, or other processes that may support mineralized tissue formation at the material-dentin interface. Retreatability refers to the ability to remove existing obturation materials sufficiently to permit further cleaning and shaping when primary endodontic treatment fails. These properties are interrelated, and improvement in one characteristic may adversely affect another; for example, increased interfacial interaction or mineralization may improve stability while simultaneously increasing resistance to removal during retreatment.

Despite substantial development of modified sealers, an important evidence gap remains. Much of the available evidence originates from in vitro investigations using different experimental models, concentrations, microbial strains, storage media, and measurement techniques. Animal and human clinical studies are comparatively fewer, and long-term clinical evidence for many modified formulations remains limited. Therefore, improvements in antimicrobial activity, ion release, or laboratory sealing performance cannot necessarily be interpreted as evidence of superior long-term clinical outcomes.

## Review

Search strategy

A structured literature search was conducted in PubMed/MEDLINE, Scopus, and Google Scholar from January 2002 through July 12, 2026. The search terms were “root canal sealer,” “endodontic sealer,” “modified root canal sealer,” “bioceramic sealer,” “nanoparticle sealer,” “quaternary ammonium endodontics,” “calcium silicate sealer,” “antimicrobial endodontic sealer,” and “sealer physicochemical properties,” used individually and in combination. The lower date limit was selected to focus on contemporary sealer formulations while retaining foundational comparative studies of sealer cytotoxicity and solubility published in 2002. Studies were included if they directly evaluated compositional modification of an endodontic sealer or reported relevant physicochemical, antimicrobial, biological, or clinical outcomes. Articles addressing unmodified materials without relevance to sealer modification, book chapters, conference abstracts, unpublished data, and case reports were excluded. Reference lists of eligible articles were screened for additional sources. Fifty-one sources were included in this comprehensive review. No formal risk-of-bias or certainty-of-evidence assessment was performed. This article was designed as a narrative comprehensive review rather than a systematic review or meta-analysis. The objective was to synthesize and critically discuss the development of modified root canal sealers across different material chemistries and modification strategies, with emphasis on antimicrobial activity, bioactivity, physicochemical performance, biological compatibility, handling characteristics, and clinical outcomes. Accordingly, the evidence was synthesized qualitatively rather than through quantitative pooling of treatment effects.

Rationale for modification of root canal sealers

Recent advances have focused on improving biological compatibility and functional performance through compositional modification. Calcium silicate-based and bioceramic sealers have attracted attention because of their generally favorable biocompatibility, potential for apatite formation, and capacity to support mineralization. Premixed calcium silicate sealers have shown lower cytotoxicity and improved histocompatibility than AH Plus in laboratory and animal studies, supporting further investigation of bioactive formulations [5]. Additives such as bioactive glass, nanoparticles, and antimicrobial agents may also modify mechanical behavior, solubility, sealing, and antibacterial activity, although the magnitude and direction of these effects depend on the formulation [6].

Modification is also driven by clinical considerations such as retreatability, dimensional stability, and compatibility with different obturation techniques. Hydraulic sealers may demonstrate favorable adaptation and dentin interaction, but these same properties can complicate removal during retreatment [7]. Differences in sealer composition affect void formation, interfacial adaptation, and sealing performance, reinforcing the need for formulations that balance biological activity, physical stability, and retrievability [8].

Overall, sealer modification aims to improve sealing, antimicrobial activity, bioactivity, handling, and biological safety without compromising dimensional stability or retreatability. Current evidence supports continued development, but no formulation consistently optimizes all of these properties.

Conventional root canal sealers: composition and limitations

Root canal sealers complement the core obturation material, typically gutta-percha, by filling the space between the filling material and the canal walls, thereby creating a three-dimensional seal throughout the root canal system. They fill anatomic irregularities, improve adaptation to dentin, and reduce bacterial recontamination after chemomechanical cleaning [9]. Conventional sealers are classified by chemistry into ZOE, epoxy resin, calcium hydroxide, and glass ionomer groups. Although conventional classifications commonly distinguish ZOE, epoxy resin, calcium hydroxide, and glass ionomer-based sealers, contemporary endodontic materials encompass additional chemistries and subgroups. These include calcium silicate-based or hydraulic sealers, bioceramic formulations, silicone-based sealers, methacrylate resin-based sealers, and experimental polymer-based formulations. Some newer materials also combine different functional components within the same matrix, such as calcium silicate with bioactive fillers, resin matrices with quaternary ammonium compounds or nanosilver, and multifunctional formulations containing both antimicrobial and remineralizing agents. Because the present review focuses specifically on modifications intended to improve the biological, antimicrobial, physicochemical, or handling properties of root canal sealers, these newer materials are considered according to their modification strategy rather than being restricted to a single conventional chemical classification. Although each has served clinicians reliably, every category carries constraints that influence seal integrity and healing over time [10].

ZOE Sealers

ZOE sealers combine zinc oxide powder with an eugenol liquid and set through a chelation reaction. Advantages include straightforward mixing, an initial antimicrobial effect from free eugenol, a long clinical record, and resorbability of extruded material. The principal limitation is high solubility in tissue fluids, which promotes washout, porosity, and gradual shrinkage over time. Eugenol release may also cause transient tissue irritation and cytotoxicity [11]. Reformulations incorporating calcium phosphate or anti-inflammatory agents aim to reduce discomfort, but solubility continues to limit long-term performance.

Epoxy Resin-Based Sealers

Epoxy formulations such as AH Plus combine bisphenol A diglycidyl ether with amine hardeners and radiopacifiers, forming a cross-linked matrix during polymerization. They offer good flow, deep adaptation to dentin, low solubility, and dimensional stability, and perform well with lateral compaction and warm vertical techniques. Limitations include polymerization shrinkage that can create interfacial gaps, mild cytotoxicity from residual monomers, and limited bioactivity [3]. Newer iterations aim to reduce shrinkage and improve tissue tolerance while retaining mechanical strength.

Calcium Hydroxide-Based Sealers

These materials disperse calcium hydroxide within a resin or salicylate matrix with radiopacifiers, releasing hydroxyl ions through an acid-base reaction to produce an alkaline environment. The resulting high pH provides antimicrobial activity and supports tissue repair, making these sealers suitable for necrotic pulps and chronic apical conditions. Their main drawbacks are notable solubility, which reduces volume and sealing durability over time, and comparatively low mechanical strength [2]. Recent formulations add resins or stabilizers to slow dissolution, with mixed results.

Glass Ionomer-Based Sealers

Glass ionomer sealers combine fluoroaluminosilicate glass with polyacrylic acid and set through an acid-base reaction that bonds chemically to dentin while releasing fluoride. Moisture sensitivity during setting produces inconsistent outcomes, solubility and dimensional change can compromise durability [12], and weak bonding to gutta-percha increases failure risk. Resin-modified versions reduce moisture sensitivity, but adoption remains slower than for other sealer types. The composition and qualitative characteristics of conventional root canal sealers is shown in Table 1.

**Table 1:** Composition and qualitative characteristics of conventional root canal sealers Summary based on references [2,3,9-14]. BaSO₄, barium sulfate; Ca(OH)₂, calcium hydroxide; ZnO, zinc oxide; ZrO₂, zirconium oxide

Sealer	Principal components	Setting mechanism	Potential benefits	Important drawbacks
Zinc oxide-eugenol	ZnO, eugenol, and additives such as rosin and BaSO₄	Chelation reaction	Initial antimicrobial activity; familiar handling; long clinical history; extruded material may resorb	Relatively high solubility; dimensional change; transient eugenol-related cytotoxicity
Epoxy resin-based	Epoxy resin, amines, and radiopacifiers such as ZrO₂	Polymerization	Generally favorable flow and low solubility; good dentin adaptation	Polymerization shrinkage or interfacial gaps; residual-monomer cytotoxicity; limited bioactivity; retreatability may be difficult
Calcium hydroxide-based	Ca(OH)₂, resin or salicylate matrix, and radiopacifiers	Acid-base or resin-setting reaction	Alkaline pH; antimicrobial and reparative potential	Variable to high solubility; comparatively limited long-term durability
Glass ionomer-based	Fluoroaluminosilicate glass and polyacrylic acid	Acid-base reaction	Chemical interaction with dentin; fluoride release	Moisture-sensitive setting; variable solubility and sealing; limited interaction with gutta-percha

Physicochemical and handling characteristics

Sealer performance depends on radiopacity, solubility, dimensional stability, interfacial adaptation, wettability, dentinal tubule penetration, and retreatability. ZOE sealers generally provide adequate initial flow but show comparatively high solubility and dimensional change. Epoxy resin sealers commonly demonstrate favorable radiopacity, low solubility, and deep tubule penetration, although their set resin matrix may complicate removal. Calcium hydroxide-based sealers show alkaline activity but variable solubility and durability. Glass ionomer sealers can interact chemically with dentin, but their performance is sensitive to moisture and formulation [8]. A qualitative comparison of selected physicochemical properties is detailed in Table 2.

**Table 2:** Qualitative comparison of selected physicochemical properties Terms are intentionally qualitative because results vary by formulation and test method. Summary based on references [2,8-14]. Ca(OH)₂, calcium hydroxide; ZOE, zinc oxide-eugenol

Property	ZOE	Epoxy resin	Ca(OH)₂	Glass ionomer
Radiopacity	Formulation-dependent; generally adequate	Generally high	Generally adequate	Formulation-dependent
Solubility	Generally high	Generally low	Variable to high	Variable
Dimensional stability	Variable; shrinkage may occur	Generally favorable, with possible polymerization contraction	Variable	Moisture- and formulation-dependent
Bonding/adaptation	Primarily mechanical	Often comparatively high	Variable	Chemical interaction with dentin; magnitude variable
Tubule penetration	Variable	Often high	Variable	Moisture-dependent
Retreatability	Generally more accessible	Comparatively difficult; complete removal remains challenging	Generally more accessible	Variable

Biological properties

Biological behavior governs cytotoxicity, stem cell interaction, osteogenic potential, immunomodulation, and periapical healing. ZOE shows early eugenol-related cytotoxicity, limited stem cell support, and variable healing. Epoxy resin presents moderate early cytotoxicity offset by dependable sealing that supports healing. Calcium hydroxide produces antimicrobial and reparative effects through pH elevation, with lower long-term toxicity. Glass ionomer offers acceptable biocompatibility and fluoride benefit when moisture is controlled [13].

Conventional sealers deliver acceptable results in many situations but fall short of ideal sealing, long-term stability, sustained antimicrobial activity, biocompatibility, and ease of retreatment, largely because of shared problems of solubility and technique sensitivity. These shortcomings continue to drive material development, although conventional sealers remain a foundational option in routine practice [14].

Types of modifications in root canal sealers

Modification of root canal sealers has followed several complementary approaches, with the primary objective of addressing specific shortcomings of conventional materials rather than creating a universally superior formulation. Current reformulations can broadly be categorized according to their principal function, including enhancement of antimicrobial activity, promotion of mineralization and bioactivity, improvement of physicochemical and dimensional properties, and optimization of handling and biological compatibility. Importantly, many experimental formulations combine more than one strategy, such as antimicrobial agents with calcium- and phosphate-releasing fillers. The effects of these modifications remain dependent on the type and concentration of the additive, its interaction with the sealer matrix, and the experimental or clinical environment.

Quaternary Ammonium and Nanosilver Incorporation

Seung et al. modified AH Plus with 2.5% dimethylaminododecyl methacrylate and 0.15% nanosilver (NAg). The additives slightly reduced flow, but the tested physicochemical properties remained within the applicable ANSI/ADA limits and were comparable to those of the unmodified control for solubility, dimensional change, and setting time [15].

The modified formulations reduced *E. faecalis* viability on day 1, and the quaternary-ammonium-containing groups maintained greater antibacterial activity at 7 and 14 days than unmodified AH Plus. Quaternary ammonium monomers act primarily through contact-mediated disruption of negatively charged bacterial membranes, whereas NAg can contribute ion-mediated antibacterial activity [15].

The Seung report contains inconsistent nomenclature: the full chemical name is presented as dimethylaminododecyl methacrylate, whereas the abbreviation DMAHDM and the reported 16-carbon chain length align with dimethylaminohexadecyl methacrylate. The terminology should therefore be interpreted cautiously.

Silver-Polydopamine-Modified Hydroxyapatite Fillers

Silver-polydopamine-modified hydroxyapatite (HA-PDA-Ag-PDA) core-shell fillers have been evaluated at 1 wt% and 2 wt% in a resin-based sealer. Both concentrations increased antibacterial activity against *E. faecalis*, promoted mineral deposition, and improved wettability compared with the unmodified material [16].

The antibacterial effect was attributed in part to Ag⁺ release, which can alter membrane permeability and interfere with cellular enzymes and nucleic acids. The polydopamine coating provides catechol groups that interact with dentin and bind Ca²⁺, thereby supporting hydroxyapatite nucleation. Its hydrophilicity may also improve wetting and interfacial contact; however, these findings are based primarily on in vitro testing [16].

Povidone- and Polycarboxylate-Modified Tricalcium Silicate

BioRoot RCS incorporates a water-soluble povidone liquid polymer and a polycarboxylate plasticizer in the powder to balance handling and hydration. The reported final setting time was approximately 300 minutes, and radiopacity was 5.2 mm Al. Povidone regulates early hydration and paste consistency, whereas the polycarboxylate reduces water demand and improves flow [17].

Hydration releases Ca²⁺ and OH⁻, producing an alkaline environment (approximately pH 11-12) and substantial calcium release, reported as 721 ppm at three hours. In phosphate-containing media, these ions promoted precipitation of B-type carbonated apatite on the material surface, which may support interfacial mineralization [17].

BioRoot RCS also demonstrated relatively high porosity, water sorption, and solubility in distilled water, in some tests exceeding the ISO mass-loss threshold. Its behavior was strongly medium-dependent: storage in phosphate-buffered saline promoted apatite deposition and reduced apparent material loss compared with distilled water [17].

Polyurethane Expandable Sealers

Polyurethane expandable sealers were developed to reduce interfacial gaps associated with setting contraction. Their combination of flexible and rigid polymer segments can produce controlled expansion of up to approximately 2% during curing [18,19]. This controlled expansion may enhance adaptation to the canal walls and minimize interfacial gaps; however, expansion alone is insufficient to ensure the establishment of a hermetic seal.

In laboratory comparisons, the polyurethane formulation showed greater dentinal tubule penetration and higher L929 fibroblast viability than selected comparator sealers. It also demonstrated angiogenic activity in an experimental choroidal neovascularization model, although the relevance of this finding to human periapical healing remains uncertain [20].

Pachymic Acid (PAC)-Modified Epoxy Resin

PAC, an organic triterpenoid, has been incorporated into AH Plus to reduce inflammatory and cytotoxic effects. In a randomized clinical study, the AHP/PAC group had lower postoperative pain at 24 hours than the AH Plus group (p = 0.03), while both groups were pain-free by day 7. Differences in lesion reduction were not significant at three months, but lesion size and volume were lower in the AHP/PAC group at 6, 9, and 12 months (p < 0.001), suggesting improved radiographic periapical healing over the 12-month follow-up period [21].

Laboratory studies indicate that PAC can reduce reactive oxygen species and inflammatory signaling in osteoblast-like cells exposed to AH Plus. Reported effects include reduced nuclear factor kappa B signaling and lower expression of nitric oxide, tumor necrosis factor-alpha, interleukin-1 beta, RANKL, COX-2, MMP-2, and MMP-9. PAC also reduced the cytotoxicity of several tested sealers in vitro [22,23].

Dimethylaminohexadecyl Methacrylate and Nanoparticles of Amorphous Calcium Phosphate (NACP)

A dual-component experimental sealer containing 5% dimethylaminohexadecyl methacrylate and 20% NACP was designed to combine contact-mediated antibacterial activity with calcium and phosphate ion release. The formulation had a flow of 28.99 ± 0.69 mm and did not differ significantly from commercial controls in the tested sealing model (p > 0.05) [24].

The 5% quaternary ammonium component reduced mature *E. faecalis* biofilm colony-forming units by more than four orders of magnitude compared with AH Plus and the unmodified experimental control. The 20% NACP component released calcium and phosphate ions and may support remineralization of adjacent dentin [24].

Triple-Agent Antibacterial and Mineralizing Modification

A triple-component experimental sealer combined 5% dimethylaminohexadecyl methacrylate, 0.15% NAg, and 30% NACP. Flow and film thickness remained within the tested standards. The NACP fraction neutralized acidic conditions and released calcium and phosphate ions. After irrigation-related dentin softening, microhardness in the treated group was not statistically different from that of sound, unirrigated dentin; this finding should not be interpreted as formal equivalence [25].

The combined quaternary ammonium and nanosilver components reduced viable *E. faecalis* biofilm counts by approximately three orders of magnitude compared with unmodified controls. The results demonstrate complementary contact-mediated and ion-mediated antibacterial activity in vitro [25].

Handling characteristics of modified root canal sealers

Clinical use depends on a sealer's handling characteristics as well as biological safety. Monocalcium silicate-based sealers have shown favorable biocompatibility and mineralization potential, but changes in powder-to-liquid ratio or the addition of accelerators can alter flow and hydration even when measured properties remain within regulatory ranges [26].

The handling, adaptation, and physical behavior of hydraulic calcium silicate sealers are closely linked to matrix design, environmental moisture, and setting chemistry [27]. Modifications intended to improve mechanical or biological performance therefore require testing to ensure that clinically important handling properties are preserved.

Flow

A root canal sealer requires sufficient flow to enter anatomic irregularities without creating an excessive risk of apical extrusion. ISO 6876:2025 requires a flow diameter of at least 17 mm for Type 1 sealing materials [28], whereas a 20-mm minimum has traditionally been associated with ANSI/ADA specifications. Experimental MTA-salicylate resin formulations illustrate how matrix composition and filler loading can be adjusted to obtain clinically acceptable flow [29]. Excessive viscosity may limit penetration into lateral anatomy, while excessive fluidity may increase extrusion and reduce dimensional stability.

Film Thickness

Film thickness influences whether a sealer forms a thin, continuous layer between gutta-percha and dentin. Epoxy resin and bioceramic sealers differ in film thickness and dimensional behavior because of their distinct matrices and setting reactions [30]. A thinner, continuous film may reduce the volume of sealer susceptible to defects, but changes made to improve flow should be evaluated to ensure that they do not increase interfacial thickness or compromise stability.

Working Time and Solubility

Working time defines the period during which a sealer can be manipulated before irreversible setting. Epoxy resin, ZOE, calcium hydroxide, and bioceramic sealers show different handling windows because of their reaction kinetics and matrix composition [31]. These characteristics also affect solubility: conventional polymeric sealers generally show limited mass loss, whereas hydraulic calcium silicate sealers may demonstrate greater early ion leaching. Modern formulations must balance a practical working time, controlled solubility, and therapeutic ion release.

Setting Time

Setting time determines how rapidly a sealer changes from a workable paste to a stable solid. Montmorillonite nanoclay can alter epoxy polymerization and shorten setting time while maintaining tested ISO requirements [32]. Similarly, hydroxyapatite produces concentration-dependent effects in hydraulic calcium silicate formulations: moderate additions may improve early properties, whereas higher concentrations can accelerate hydration and shorten working time [33]. Both excessively rapid and markedly delayed setting may complicate clinical use.

Radiopacity

Radiopacity is required for postoperative assessment of filling extent and voids. ISO 6876 and ANSI/ADA Standard No. 57 require a 1-mm specimen to have radiopacity equivalent to at least 3 mm of aluminum. ProRoot MTA has been reported to show 4.32 ± 0.17 mm Al because of its bismuth oxide content, whereas Biodentine showed 2.29 ± 0.21 mm Al in one study, below the specified threshold [34]. Alternative radiopacifiers such as tantalum oxide and zirconium oxide are used to reduce discoloration associated with bismuth oxide.

Dimensional Stability

Dimensional stability influences maintenance of the sealer-dentin and sealer-core interfaces. Epoxy resin sealers may undergo limited polymerization contraction, whereas hydraulic materials can show volume changes after hydration and immersion. Kwak et al. reported measurable volumetric shrinkage in several calcium silicate-based sealers after 30 days in distilled water [35]. Although the tested materials met the dimensional-change criterion used in that study, the observed contraction indicates that adaptation should not be inferred from standard compliance alone.

Bond Strength

Push-out bond strength testing by Shieh et al. showed a higher mean value for AH Plus Jet (3.26 ± 1.06 MPa) than for EndoSequence BC Sealer (2.38 ± 0.75 MPa) and AH Plus Bioceramic Sealer (2.16 ± 0.72 MPa). Cohesive failure was more common with the epoxy resin material, whereas adhesive failure at the dentin interface occurred more often with the bioceramic sealers [36]. These findings indicate that bond strength depends on matrix chemistry and interfacial interaction, not only apparent adaptation.

Dentinal Tubule Penetration and Resistance to Displacement

Wettability, particle size, matrix chemistry, and dentin condition influence tubule penetration and resistance to displacement. Verma et al. reported higher push-out values for AH Plus than for BIO-C ION+ and NanoSeal-S across root thirds [37]. The findings were formulation-specific and should not be generalized to all resin, calcium silicate, or silicone sealers.

BIO-C ION+ showed the greatest mean penetration depth in that study, including 1184.69 µm coronally, compared with 864.14 µm for AH Plus and 495.64 µm for NanoSeal-S. Penetration decreased apically in all groups, consistent with lower tubule density, greater sclerosis, and less effective smear-layer removal near the apex [37].

Retreatability

Retreatability is important because deep interfacial penetration may complicate secondary treatment. Solvents can soften gutta-percha and selected resin components; however, complete removal of epoxy resin sealers from dentinal tubules and canal irregularities remains challenging.

In contrast, hydrated calcium silicate sealers form rigid calcium silicate hydrate phases and a mineral-rich interfacial zone that is not predictably dissolved by conventional endodontic solvents. Mechanical instrumentation is therefore central to removal. A scanning electron microscopy study found residual material after retreatment of canals filled with either bioceramic or epoxy resin sealers, although working length and apical patency could be re-established in the tested specimens [38]. A single laboratory study does not establish that retreatment outcomes are universally predictable.

Biological and antimicrobial properties of modified sealers

Biofunctionalization strategies aim to combine antibacterial activity with acceptable cytocompatibility and physical behavior. This is clinically relevant because bacteria may persist in dentinal tubules, isthmuses, and established *E. faecalis* biofilms after chemomechanical preparation. An experimental methacrylate-resin sealer containing 0.15% NAg and 5% dimethylaminohexadecyl methacrylate produced an approximately 6-log reduction in *E. faecalis* colony-forming units compared with unmodified controls while meeting the tested flow and film-thickness requirements [39].

A systematic review concluded that incorporating nanoparticles into sealer matrices generally increases antimicrobial activity against planktonic organisms and biofilms, although study methods and formulations were heterogeneous [40]. Silver, chitosan, and hydroxyapatite nanoparticles have also been tested in bioceramic sealers. In one study, nanoparticle modification increased antibacterial activity and push-out bond strength and reduced solubility in the apical third; these findings were formulation-specific [41].

Chlorhexidine-hexametaphosphate nanoparticles measuring approximately 23-53 nm have been incorporated at 2% and 5% by weight into AH Plus, MTA Fillapex, and Pulp Canal Sealer. The modified materials showed greater antimicrobial activity than their unmodified counterparts. Radiopacity was unchanged, while flow decreased and 24-hour solubility increased; most measured values remained within the applicable limits [42].

Drug-silica coassembled particles loaded with octenidine dihydrochloride have also been evaluated. At 1% and 2% by weight in EndoSequence BC Sealer, octenidine release remained above the minimum inhibitory concentration for *E. faecalis* for 30 days, and antibacterial activity persisted throughout the test period. The effect was smaller in AH Plus, suggesting that the carrier matrix matters as much as the antimicrobial additive because it governs drug release [43].

Quaternary ammonium polyethylenimine nanoparticles at 1%-2% by weight increased the sustained contact-killing activity of experimental sealers against *E. faecalis *[44]. Although this study evaluated acrylic repair resin rather than a root canal sealer, its findings provide supplementary evidence regarding the concentration-dependent behavior of dimethylaminohexadecyl methacrylate in a related dental-material system. These findings should therefore not be interpreted as direct evidence of the performance of DMAHDM-modified root canal sealers [45]. Although that study evaluated acrylic repair resin rather than a root canal sealer, it supports the concentration-dependent behavior of the monomer.

Overall, laboratory evidence indicates that nanoparticles and quaternary ammonium compounds can increase antibacterial activity while preserving selected physical properties in some formulations. These findings require standardized cytocompatibility testing and prospective clinical validation before they can be translated into claims of improved healing or reduced retreatment. 

The available evidence should be interpreted according to study design. Most investigations of modified root canal sealers remain laboratory-based and evaluate isolated properties such as antimicrobial activity, ion release, flow, solubility, bond strength, or dentinal tubule penetration. These studies are useful for identifying material mechanisms and screening promising formulations but do not establish clinical effectiveness. Animal studies provide additional information regarding biological compatibility and tissue response, whereas human clinical studies provide the most direct evidence of treatment outcomes. Because relatively few modified formulations have undergone adequately powered, long-term clinical evaluation, laboratory improvements should not be interpreted as evidence of established clinical superiority.

Clinical implications and future directions

Clinical Implications

The clinical relevance of sealer modification can be considered across periapical healing, postoperative symptoms, bacterial control, handling, and retreatability [46]. Calcium silicate-based sealers demonstrate favorable biological properties and encouraging clinical outcomes; however, consistent clinical superiority over established epoxy resin sealers has not been confirmed [46,47]. Their potential to support hard-tissue repair is generally attributed to calcium ion release, alkalinity, and apatite formation at the dentin-material interface [47]. These mechanisms should be distinguished from direct antimicrobial drug release.

In an experimental in vivo model, a magnetic nanoparticle-modified sealer reduced persistent apical periodontitis compared with an unmodified control [48]. The result suggests that magnetic nanoparticles may improve antibacterial delivery or tubule penetration, but it should not be presented as an established clinical association. Human clinical studies are required to determine whether the effect is reproducible and clinically meaningful.

Bioactive matrices and antimicrobial nanoparticles may therefore be complementary, but direct evidence evaluating combined formulations remains limited. Their simultaneous use should be investigated with designs that isolate the contribution of each modification.

Technique-related modifications may also influence obturation quality. A modified methacrylate-based sealer used with a single-cone technique showed improved filling characteristics and fewer voids in the tested model [49]. This finding supports further evaluation of material designs intended for simplified obturation protocols, but it does not establish equivalence across all canal anatomies or operators.

Retreatability remains unresolved. Calcium silicate-containing sealers may require longer removal times and may leave more residual material than epoxy resin sealers [50]. The hydration products and dentin interaction that contribute to bioactivity can also increase resistance to solvents and instrumentation. No available formulation has demonstrated consistently superior performance across bioactivity, antimicrobial penetration, handling, and retrievability.

Clinical Studies

A 2018 retrospective cohort study evaluated the clinical outcomes of nonsurgical root canal treatment using EndoSequence BC Sealer with a single-cone obturation technique in 307 teeth with an average follow-up of 30.1 months. The overall success rate was 90.9%, with significantly higher success observed in cases with periapical lesions <5 mm compared with larger lesions. Sealer extrusion occurred in 47.4% of cases but did not significantly influence treatment outcomes, supporting BC Sealer as a viable option for root canal obturation [51]. In A 2020 non-randomized clinical study, root canal treatment using a calcium silicate sealer (BioRoot™ RCS) with a single-cone technique achieved 84% success on CBCT and 90% success on periapical radiographs at 12 months, compared with 80% and 89%, respectively, for AH Plus used with warm vertical condensation. As no statistically significant difference was observed between the groups (p = 0.6099), the calcium silicate sealer/single-cone technique appears to be a clinically effective alternative to conventional warm vertical condensation [52]. A 2022 randomized controlled clinical trial compared calcium silicate-based sealers (CeraSeal and EndoSeal TCS) with epoxy resin-based sealers (AH Plus and ADseal) in 71 analyzed teeth over a three-month follow-up period. No significant differences were observed among the sealers regarding obturation quality (voids and sealer extrusion; p > 0.05) or postoperative pain, and 70 of 71 teeth (98.6%) showed successful outcomes. The study concluded that calcium silicate-based and epoxy resin-based sealers provide comparable short-term clinical outcomes, with variations in filling quality likely related to the specific properties of each sealer [53]. A 2025 multicentric randomized controlled non-inferiority trial evaluated the 24-month clinical efficacy and safety of injectable calcium silicate sealer (BioRoot™ Flow) compared with the hand-mixed BioRoot™ RCS in root canal treatment. Among 160 treated patients, the 24-month success rates were 86.6% for BioRoot Flow and 87.7% for BioRoot RCS (p = 0.0195), demonstrating non-inferiority of the injectable formulation. No significant differences were observed in secondary outcomes, including obturation quality, postoperative pain, material resorption, or adverse events, supporting BioRoot Flow as a safe and effective alternative for clinical use [54].

Taken together, the available clinical evidence suggests that calcium silicate-based sealers can provide clinically acceptable short- and medium-term outcomes, but current evidence does not establish consistent superiority over established epoxy resin-based sealers. Differences among studies in obturation technique, lesion characteristics, sealer formulation, follow-up duration, and outcome assessment limit direct comparison. Importantly, the strongest clinical evidence currently concerns commercially available calcium silicate-based sealers, whereas many of the experimental antimicrobial and multifunctional reformulations discussed earlier remain at the laboratory stage. Therefore, the promising biological and physicochemical characteristics of experimental modifications should not be extrapolated to clinical effectiveness until supported by appropriately designed human studies.

Future Research Directions

Comparative studies that isolate a single design variable are uncommon; most compare materials that differ simultaneously in base chemistry, particle size, filler loading, and setting mechanism. Future research should move beyond incremental changes and evaluate multifunctional platforms using standardized outcomes.

Smart and stimuli-responsive sealers could alter ion release, pH, or antimicrobial activity in response to local environmental conditions rather than releasing agents at a constant rate. Controlled drug-delivery systems incorporating antimicrobials, anti-inflammatory agents, or growth factors may extend biological activity after placement, but release kinetics and cytotoxicity require careful evaluation.

Regenerative endodontic applications may require sealers that support tissue ingrowth rather than simply provide obturation. Artificial intelligence and machine-learning methods may also assist in screening filler ratios, particle morphology, and setting kinetics before laboratory synthesis. These approaches remain exploratory and require experimental validation.

Material selection may ultimately become more case-specific, taking into account lesion size, canal anatomy, moisture conditions, and the likelihood of retreatment. Progress will depend on multicenter randomized controlled trials with standardized clinical, radiographic, microbiological, and patient-reported outcomes [51].​​​​​​​

Limitations of the review

Several limitations of this comprehensive review warrant acknowledgment. The absence of a registered search protocol and formal quality-of-evidence grading introduces selection bias inherent to the review design. The included studies are markedly heterogeneous in experimental model (in vitro, in vivo, and clinical), sealer base chemistry, particle size and concentration, and outcome measurement, precluding quantitative pooling or direct comparison of effect sizes. The preponderance of short-term in vitro and single-institution data may not accurately reflect long-term behavior in the complex biological milieu of the human root canal system. Clinical trial data remain sparse, follow-up periods in available randomized trials are short relative to the longevity expected of endodontic treatment, and publication bias toward positive results may overrepresent the efficacy of the modifications reviewed. 

As this was a narrative comprehensive review, study selection and synthesis were not conducted using a registered systematic-review protocol or a formal duplicate-independent screening and risk-of-bias framework. Consequently, the possibility of selection and interpretation bias cannot be excluded. The findings should therefore be regarded as a structured synthesis of the available literature rather than as a systematic estimate of comparative treatment effects. Future systematic reviews should use predefined protocols, database-specific reproducible search strategies, independent screening and data extraction, formal risk-of-bias assessment, and standardized evidence grading to determine the comparative effectiveness of modified root canal sealers.

## Conclusions

Modification of root canal sealers with bioactive fillers, nanoparticles, calcium-releasing compounds, and quaternary ammonium agents has produced promising improvements in antimicrobial activity, ion release, remineralization potential, and selected biological properties. However, these benefits are material- and concentration-dependent and may be accompanied by increased solubility, altered handling, cytotoxicity, or reduced retreatability. Multicenter clinical trials with standardized outcome measures are needed to confirm the long-term performance of these modified sealers across diverse clinical conditions.
